# Stretchable and Shape-Transformable Organohydrogel with Gallium Mesh Frame

**DOI:** 10.3390/gels10120769

**Published:** 2024-11-26

**Authors:** Mincheol Lee, Youngjin Choi, Young Min Bae, Seonghyeon Nam, Kiyoung Shin

**Affiliations:** 1 Electro-Medical Equipment Research Division, Korea Electrotechnology Research Institute (KERI), Ansan 15588, Republic of Korea; 2 School of Chemical and Biological Engineering, Institute of Chemical Processes, Seoul National University, Seoul 08826, Republic of Korea; 3 Center for Nanoparticle Research, Institute for Basic Science (IBS), Seoul 08826, Republic of Korea

**Keywords:** stretchable hydrogel, shape-transformable hydrogel, liquid metal, shape memory

## Abstract

Shape-memory materials are widely utilized in biomedical devices and tissue engineering, particularly for their ability to undergo predefined shape changes in response to external stimuli. In this study, a shape-transformable organohydrogel was developed by incorporating a gallium mesh into a polyacrylamide/alginate/glycerol matrix. The gallium mesh, which transitions between solid and liquid states at moderate temperatures (~29.8 °C), enhanced the hydrogel’s mechanical properties and enabled shape-memory functionality. The composite organohydrogel exhibited a high elastic modulus of ~900 kPa in the solid gallium state and ~30 kPa in the liquid gallium state, enabling reversible deformation and structural stability. Glycerol improved the hydrogel’s moisture retention, maintaining stretchability and repeated heating and cooling cycles. After multiple cycles of the shape-changing process, the organohydrogel retained its mechanical integrity, achieving shape-fixation and recovery ratios of ~96% and 95%, respectively. This combination of shape-memory functionality, stretchability, and mechanical stability makes this organohydrogel highly suitable for applications in flexible electronics, soft robotics, and biomedical devices, where adaptability and shape retention are essential.

## 1. Introduction

In recent years, there has been growing interest in shape-memory materials for their potential in biomedical devices and tissue engineering, particularly for their ability to undergo predefined shape changes in response to specific stimuli, such as temperature, pH, and light [1,2,3]. Shape-memory polymers (SMPs) have been explored extensively for their adaptability to specific defect sites and their potential to be used in minimally invasive procedures. These materials combine flexibility, mechanical strength, and controlled shape retention, making them ideal for advanced biomedical applications. At the same time, hydrogels have emerged as a critical class of materials in areas such as tissue engineering, drug delivery, and soft robotics, due to their high flexibility, biocompatibility, and tunable mechanical properties [4,5,6]. Composed primarily of water and crosslinked polymer networks, hydrogels closely mimic the physical properties of natural tissues, making them ideal for biomedical applications [7,8,9]. The ability of hydrogels to conform to irregular surfaces and respond dynamically to environmental changes further enhances their versatility. Integrating shape-memory capabilities into hydrogels could significantly expand their application potential, leveraging their native flexibility and biocompatibility while introducing controlled responsive shape transformations for use in a wider range of medical and engineering fields [10,11,12].

A commonly studied stimulus for shape-memory hydrogels is temperature [11,13,14,15], due to the temperature differences between the body and external environments, making it a practical cue for triggering shape transformation. There are various methods to achieve shape-memory functionality in hydrogels, such as using temporary crosslinks and network architectures [16,17,18,19]. However, the type of hydrogel required often varies depending on the application. This creates a need for a universal approach that can be applied across different fields and environments. One prominent approach to creating shape-memory hydrogels involves the incorporation of shape-memory polyurethanes (SMPU) [20], which provide temperature responsiveness and excellent shape retention properties. While this method offers significant advantages in terms of shape memory functionality, it tends to have limitations in terms of stretchability, which can restrict its application in environments that require high flexibility or large deformations.

**Figure 1:**
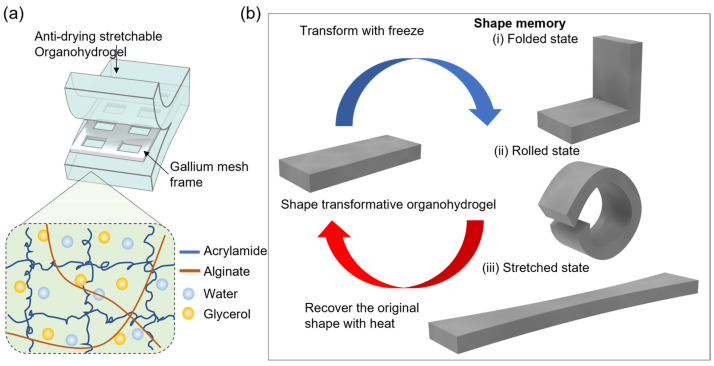
Overall schematic of the shape-transformable organohydrogel. (**a**) The structure of the shape-transformable organohydrogel. (**b**) The shape transformation cycle of the organohydrogel illustrating the transition between the original and deformed states.

In this work, we propose a new approach to providing shape-memory capabilities to hydrogels by incorporating liquid metal, specifically gallium, into the hydrogel matrix (Figure 1a). Gallium, which transitions between solid and liquid states at relatively low temperatures (~29.8 °C), provides mechanical reinforcement without sacrificing the stretchability of the hydrogel. This approach builds on recent studies that used gallium in flexible devices for tunable stiffness [21,22,23,24].

The hydrogel matrix was designed using polyacrylamide (PAAm) and alginate, selected for their ability to combine mechanical strength and stretchability [25,26]. Together, PAAm and alginate form a semi-interpenetrating polymer network, providing a stable and robust platform for embedding the gallium mesh.

By embedding a gallium mesh within the stretchable hydrogel, we aim to overcome the handling challenges typically associated with the low modulus of hydrogels, while allowing the material to be deformed, stretched, or compressed when required. As shown in Figure 1b, the gallium mesh solidifies upon cooling to provide elasticity and mechanical strength but liquefies under heat, allowing the hydrogel to return to its original shape by releasing the stored stress within the polymer matrix. Furthermore, we incorporated glycerol into the hydrogel matrix to maintain its stretchability and flexibility during heating and cooling cycles. Glycerol’s hygroscopic nature helps to prevent drying, which is crucial for ensuring the hydrogel remains functional and robust over extended periods of use [15,27,28]. This unique combination of shape-memory functionality and stretchability leverages the flexibility of organohydrogels with the supportive and adaptable properties of gallium, resulting in a material that balances adaptability and mechanical stability.

This study presents a novel organohydrogel system that integrates a gallium mesh to overcome the low modulus and poor shape retention typically seen in conventional hydrogels. The addition of glycerol improves the hydrogel’s resistance to freezing and drying for repetitive shape changes, further enhancing its versatility in various environmental conditions. This universal design can be adapted to other hydrogel formulations, broadening its potential applications. This work demonstrates how hydrogels can be engineered for use in demanding mechanical and biomedical applications, combining stretchability with robust shape-memory behavior.

## 2. Results and Discussion

### 2.1. Characteristics of the Anti-Drying Stretchable Hydrogel

We selected a PAAm-alginate hydrogel as the matrix for its excellent stretchability, fracture toughness, and biocompatibility. However, the shape-changing process requires repeated heating and cooling, and for long-term use, preventing drying becomes essential. To address this, we incorporated glycerol into the hydrogel matrix due to its hygroscopic nature, which helps improve moisture retention and acts as a plasticizer, enhancing polymer chain mobility. In this section, we evaluate the impact of varying glycerol concentrations (0–50 wt% of the hydrogel precursor solution) on the organohydrogel’s drying behavior, mechanical properties, and stress relaxation.

**Figure 2:**
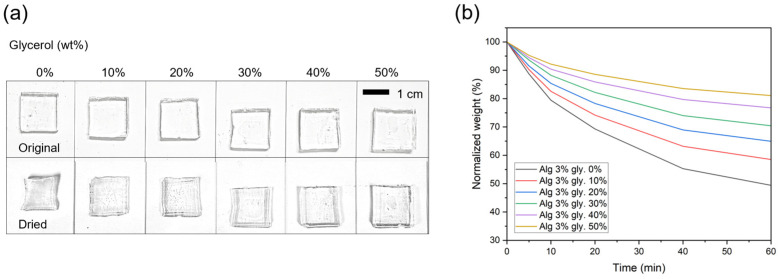
Effect of glycerol concentration on the anti-drying properties of organohydrogels. (**a**) Visual comparison of organohydrogel samples with varying glycerol concentrations (0–50 wt% of aqueous solution) before and after a drying process. The top row shows the original organohydrogel samples, while the bottom row shows the samples after drying. (**b**) Normalized weight loss of organohydrogels over time in a 60 °C convection oven.

The anti-drying properties of the organohydrogels were analyzed by measuring weight loss during drying at 60 °C over time (Figure 2). Figure 2a shows the visual comparison of organohydrogel samples before and after the drying process. As the glycerol concentration increases, the organohydrogel exhibits less shrinkage and maintains their original shape more effectively. The normalized weight loss over time (Figure 2b) demonstrates that higher glycerol content slows down the rate of water loss. At higher glycerol concentrations, particularly at 30% and above, the organohydrogels demonstrated significantly improved moisture retention compared to those with lower glycerol content. The organohydrogel with 30% glycerol retained approximately 70% of its original weight after 60 min of drying at 60 °C, indicating its potential to resist drying out in ambient conditions. This suggests that glycerol plays a crucial role in maintaining organohydrogel’s flexibility and preventing cracking or brittleness over extended periods.

**Figure 3:**
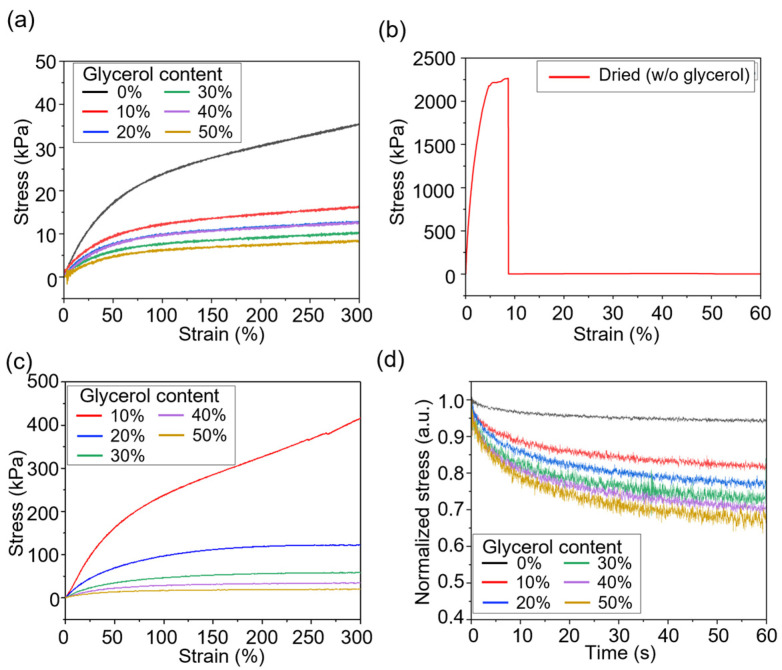
Mechanical properties of organohydrogels with varying glycerol concentrations after a drying process. (**a**) Stress–strain curves of organohydrogels with varying glycerol concentration before drying. Stress–strain curves of the organohydrogel (**b**) without glycerol and (**c**) with glycerol after the drying period. (**d**) Stress relaxation tests under 100% elongation of the organohydrogels.

The mechanical properties of the organohydrogels were evaluated after a one-week drying period at room temperature (Figure 3). Before the drying process, organohydrogels with varying glycerol concentrations exhibited high stretchability (>300%) and low modulus (<40 kPa) (Figure 3a). However, after the drying period, the hydrogel with no glycerol became too stiff to stretch due to a high modulus (>25 MPa), and it broke when subjected to stress (Figure 3b). In contrast, organohydrogels containing glycerol maintained their stretchability even after drying, with elongation exceeding 300%, although their modulus was higher compared to before the drying process (Figure 3c). Notably, organohydrogels with 30% or more glycerol still exhibited a moderate modulus, maintaining their mechanical properties even after the drying process.

Stress relaxation tests were performed to assess the organohydrogels’ ability to dissipate stress under strain (100% elongation, Figure 3d). Organohydrogels with higher glycerol content experienced greater stress relaxation, as shown by the rapid decrease in stress over time. This can be attributed to the increased polymer chain mobility facilitated by the glycerol. At 50% glycerol, the stress relaxation was particularly pronounced, with the material losing a significant proportion of its initial stress quickly. However, this high level of relaxation may be undesirable for applications requiring long-term mechanical stability.

To further understand the impact of glycerol on the organohydrogel’s properties, Fourier-transform infrared (FTIR) analysis was performed (Supplementary Figure S1). The results showed a shift in the O-H stretching peak to lower wavenumbers as glycerol concentration increased, indicating stronger hydrogen bonding within the organohydrogel [29,30]. This enhanced hydrogen contributes to the organohydrogel’s anti-drying properties. Additionally, the hydrogen bonds formed between glycerol and the polymer chain create a flexible network that helps dissipate stress [30,31], contributing to improved stress relaxation and flexibility.

These findings highlight the role of glycerol concentration in balancing the organohydrogel’s mechanical properties. Among the tested concentrations, 30% glycerol appears to provide the right balance between flexibility and mechanical strength, making it suitable for applications where both stretchability and moisture retention are crucial.

### 2.2. Fabrication of the Shape-Transformable Organohydrogel

**Figure 4:**
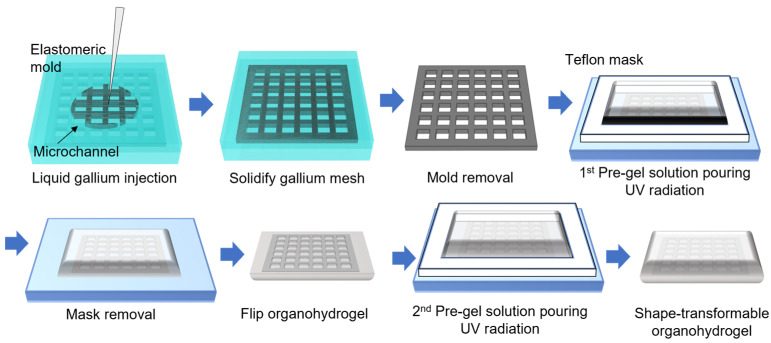
Fabrication process of the shape-transformable organohydrogel.

Figure 4 illustrates the fabrication process of the shape-transformable organohydrogel. The shape-transformable organohydrogel was fabricated by first preparing an elastomeric mold with microchannels to shape the gallium mesh (See Section 4.3). Ecoflex 0050 was selected as the elastomeric mold material due to its flexibility and stretchability, which prevent damage to the gallium mesh during the demolding process.

To address challenges with air bubble formation and ensure complete filling of the mesh structure, the gallium mesh was fabricated in two separate layers: a warp (longitudinal) layer and a weft (transverse) layer (Supplementary Figure S2). Liquid gallium was injected into these microchannels and allowed to solidify in a refrigerator (−20 °C), forming a rigid gallium layer with a thickness of 100 μm. After solidification, the two layers were carefully aligned and partially heated using a heat gun to bond them together, creating the final gallium mesh (thickness of 200 μm).

The completed gallium mesh was carefully extracted from the mold and placed on the glass substrate. A Teflon mold with a hole (thickness of 1 mm) was then placed onto the gallium mesh, and the organohydrogel pre-gel solution (See Section 4.2)—composed of acrylamide, alginate, and glycerol—was poured into the mold to encapsulate the mesh. Polymerization was carried out using UV radiation, creating a composite material where the gallium mesh provided structural support.

**Figure 5:**
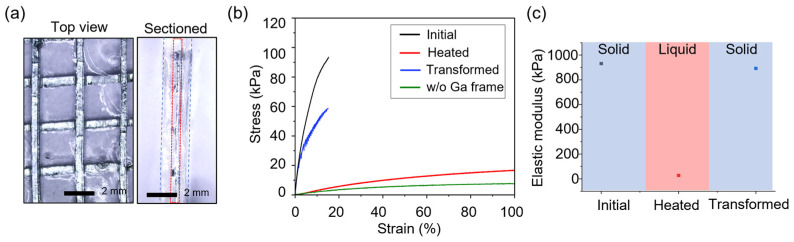
Mechanical properties of the shape-transformable organohydrogel in different states. (**a**) Microscopic images showing the gallium-reinforced organohydrogel: top view (left) and cross section (right). Red dotted box indicates the layer of the gallium mesh frame. (**b**) Stress–strain curves comparing the modulus of the organohydrogel in its initial state (black), heated state (red), and transformed state after stretching (blue) and the organohydrogel without gallium frame (green). (**c**) Elastic modulus of the shape-transformable organohydrogel in different states.

After the first layer of the organohydrogel was formed, the gel was removed from the substrate and flipped. Subsequently, another layer of the precursor solution was poured onto the opposite side and polymerized, ensuring the gallium mesh was centered within the organohydrogel. The final thickness of the gel was measured to be 2 mm (Figure 5a). This alignment positioned the mesh along the neutral mechanical plane and prevented leakage of the liquid gallium. This fabrication approach resulted in an organohydrogel that remains stretchable when the gallium is in its liquid state and exhibits enhanced mechanical stability when the gallium is in a solid state.

### 2.3. Mechanical Characterization of a Gallium-Reinforced Shape-Transformable Organohydrogel

The fabricated shape-transformable organohydrogel was assessed for its mechanical properties in various states to evaluate its ability to undergo and maintain shape transformations. Figure 5a shows microscopic images of the fabricated gallium-reinforced organohydrogel. The top view (Figure 5a, left) illustrates the structured arrangement of the gallium mesh. As shown in the image, the gallium mesh frame was generated by bonding two layers (warp and weft). The cross-section (Figure 5a, right) highlights the integration of the mesh frame within the organohydrogel matrix.

As shown in the stress–strain curves (Figure 5b), the organohydrogel reinforced with gallium exhibited significantly higher elastic modulus (E > 900 kPa, black curve) compared to the non-reinforced organohydrogel (E < 40 kPa, green curve). This demonstrates the role of the gallium mesh in enhancing the mechanical strength of the material. In its heated state (Figure 5b, red curve), where the gallium transitions to a liquid phase, the organohydrogel displays a softer stress–strain profile. Since liquid gallium does not contribute to mechanical reinforcement, the shape-transformable organohydrogel’s properties (E < 30 kPa) become similar to those of an organohydrogel without the gallium mesh.

In the transformed state, achieved by heating the organohydrogel to transition gallium to its liquid phase, stretching it (strain = 100%), and then cooling it to solidify the gallium again, we measured the stress–strain curve to further assess the material’s ability to retain mechanical integrity. Figure 5b (blue curve) shows that the transformed organohydrogel regains mechanical properties similar to its initial state, indicating that the gallium mesh enables the organohydrogel to maintain stiffness and structural stability even after undergoing deformation. When the gallium frame is in its solid state, strain exceeding 15% causes the structure to begin fracturing (Supplementary Figure S3).

Figure 5c summarizes the elastic modulus of the shape-transformable organohydrogel in different states (initial, heated, and transformed), derived from the stress–strain curves in Figure 5b. The results demonstrate that modulus significantly decreases in the heated state, enabling deformation. However, once the material is cooled, the modulus recovers, returning close to its original value, even in the transformed state. This recovery of stiffness enables the material to fix its shape and maintain structural stability in the deformed configuration.

**Figure 6:**
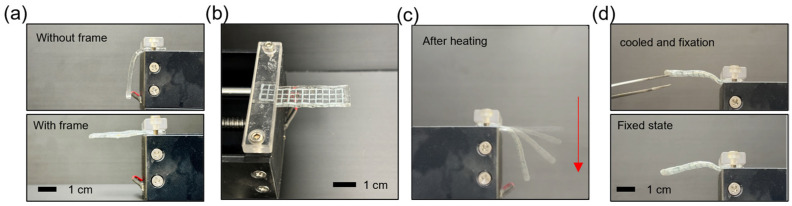
Shape transformation and fixation process of the shape-transformable organohydrogel. (**a**) Organohydrogel without (top) and with gallium mesh frame (bottom). (**b**) Top view of the organohydrogel with gallium mesh frame (**c**) Overlay image of sequential deformation of the organohydrogel with gallium mesh frame under heating. Red arrow indicates the moving direction. (**d**) Image showing the organohydrogel in its fixed state after cooling, with the shape retained from the previous deformation.

Figure 6 shows the process of shape transformation and fixation in the shape-transformable organohydrogel. The organohydrogel is shown both without the gallium frame (Figure 6a, top) and with the gallium frame (Figure 6a, bottom). The organohydrogel with the gallium mesh retains more structure compared to the organohydrogel without the mesh, demonstrating the initial mechanical support provided by the gallium reinforcement. In the next phase (Figure 6c), the organohydrogel is heated, causing the gallium to transition to a liquid state. This liquefaction allows the organohydrogel to deform under gravitational force (indicated by the arrow), illustrating its capacity to transform in response to thermal stimuli. After cooling, the organohydrogel fixes its shape in the deformed configuration, and it maintains its position without external support (Figure 6d). This confirms that the shape-transformable organohydrogel can be deformed at elevated temperatures and retain its transformed shape upon cooling.

To further evaluate the shape-fixation and recovery properties of the organohydrogel, two approaches were employed. First, a bending angle fixation test was conducted to assess the shape-transformable organohydrogel’s ability to maintain various deformed angles upon cooling (Section 4.5). Softened samples were placed in an acrylic mold to set the desired angle and then cooled to preserve the fixed state.

The shape fixation and recovery ratios for bending were calculated as follows [32,33]:

$$Shape\;fixation\;ratio=\frac{\theta_{fixed\;}}{\theta_{max}}\times 100\;$$

$$Shape\;recovery\;ratio=\frac{\theta_{max}-\theta_{i}}{\theta_{max}}\times 100\;$$

**Figure 7:**
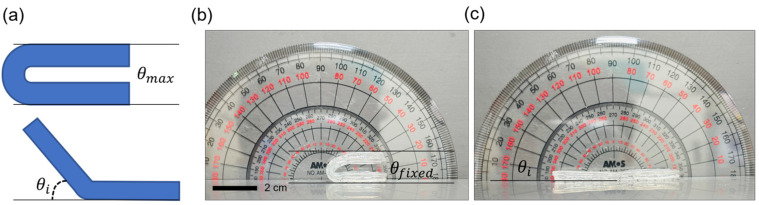
Bending angle fixation and recovery of the shape-transformable organohydrogel. (**a**) Schematic representation of the bending angle measurement for shape fixation and recovery. Maximum bending angle, including the maximum bending angle ($\theta_{max\;}$ = 180°) and the recovered angle after reheating ($\theta_{i}$). (**b**) Organohydrogel bent to a maximum angle and cooled in the mold to retain the fixed shape. (**c**) Organohydrogel returned to its original shape after reheating ($\theta_{i}$ = 0°).

**Figure 8:**
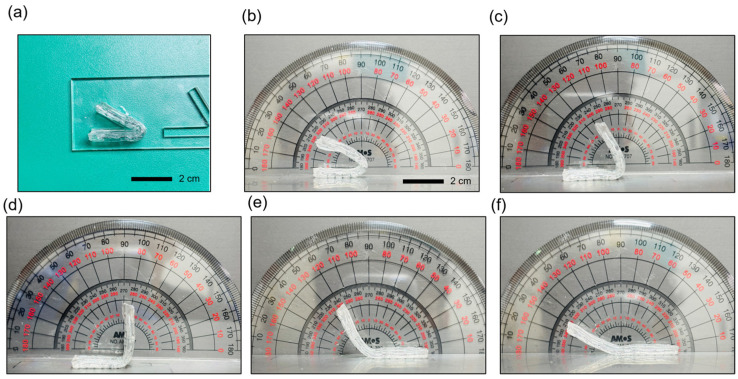
Fixed shapes of the shape-transformable organohydrogel at various bending angles. (**a**) Organohydrogel sample positioned in an acrylic mold for shape fixation (**b**–**f**) Organohydrogel fixed at bending angles of approximately 30°, 60°, 90°, 120° and 150°, respectively, demonstrating the organohydrogel’s ability to retain a range of deformed angles.

The shape fixation ratio for bending angle was observed to be nearly 100%, indicating excellent shape retention during cooling. Additionally, upon reheating, the shape recovery ratio reached 100%, demonstrating the organohydrogel’s effective return to its original shape (Figure 7). Figure 8 shows the organohydrogel fixed in various deformed angles, further illustrating its reliable shape-fixation capability across a range of bending angles.

**Figure 9:**
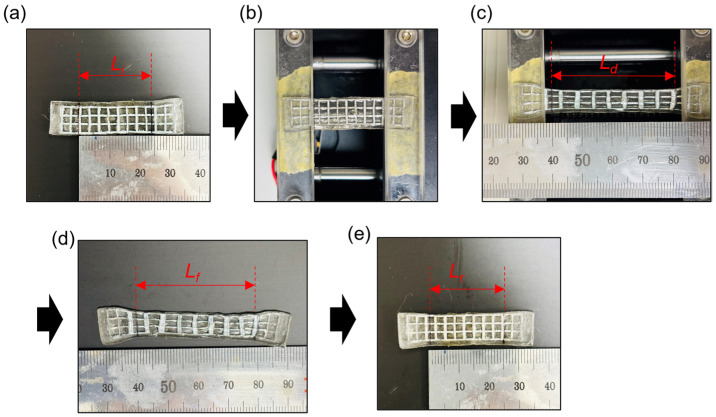
Step-by-step process for the stretching fixation test of the shape-transformable organohydrogel. (**a**) Initial length (*L_i_*) measurement of the organohydrogel sample before stretching. (**b**) Positioning of the heated sample in the stretching device. (**c**) Sample stretched to the deformed length (*L_d_*) under 100% stretching. (**d**) Fixed length (*L_f_*) measurement after cooling to maintain the deformed shape. (**e**) Recovered length (*L_r_*) measurement after reheating, illustrating the organohydrogel’s shape recovery capability.

Second, a stretching fixation test was conducted to further examine the shape-transformable organohydrogel’s shape-fixation and recovery properties under tensile deformation. Samples were heated, stretched to a specific deformation length, and then cooled to fix the stretched state (Figure 9). To ensure accurate length measurements, lines were drawn on the organohydrogel before stretching to mark specific points, as the fixed ends of the organohydrogel were also subject to elongation.

The shape fixation ratio was calculated to quantify the extent to which the organohydrogel retained its stretched length after cooling, using the following formula:

$$Shape\;fixation\;ratio\;\left(\%\right)=\frac{L_{f\;}}{L_{d}}\times 100\;$$

where *L_f_* is the length after cooling and *L_d_* is the length immediately after stretching. The shape fixation ratio was 97.6%, indicating excellent shape retention.

After fixation, the sample was reheated to assess shape recovery. The shape recovery ratio was calculated based on the recovery length (*L_r_*) after reheating, using the following formula:

$$Shape\;recovery\;ratio\;(\%)=\frac{L_{f}-L_{r}}{L_{f}-L_{i}}\times 100\;$$

where *L_i_* is the initial length of the organohydrogel before stretching. After one cycle of stretching test, the shape recovery ratio was found to be 96.9%, demonstrating the organohydrogel’s effective ability to return to its initial shape upon reheating.

**Figure 10:**
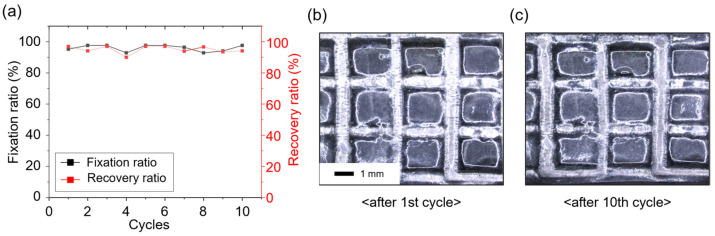
Multiple-cycle stretching fixation and recovery test results for the shape-transformable organohydrogel. (**a**) Graph showing the shape fixation and recovery ratios over multiple cycles. (**b,c**) Microscopic images of the gallium frame within the organohydrogel after multiple cycles.

A multiple-cycle stretching fixation and recovery test was conducted to evaluate the durability of the shape-transformable organohydrogel under repeated tensile deformation (Supplementary Figure S4). As shown in Figure 10a, both the shape fixation ratio and shape recovery ratio remained consistently high across multiple cycles, with the fixation ratio averaging around 96% and the recovery ratio around 95%. These results indicate the organohydrogel’s robust ability to retain and recover its deformed shape after repeated cycles, demonstrating reliable shape-memory properties. Microscopic images of the gallium frame within the organohydrogel after 10 cycles further confirm its structural integrity, showing minimal deformation in the internal structure (Figure 10b,c). The strain–stress curve indicates that after multiple cycles of deformation, the organohydrogel maintains its temperature-dependent mechanical adaptability (Supplementary Figure S5).

### 2.4. Demonstration of Shape-Transformable Organohydrogel Behavior

Building upon the previously demonstrated shape-transformable organohydrogel properties, we investigated its ability to undergo various mechanical deformations, including folding, 1D stretching, and 2D stretching, while maintaining the capacity to return to its original shape upon heating.

**Figure 11:**
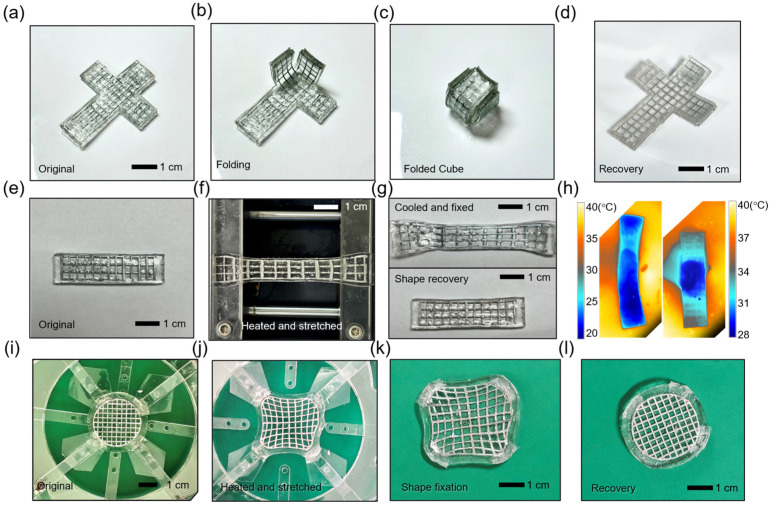
Shape transformation and fixation process of the shape-transformable organohydrogel. (**a**–**d**) folding and recovery process of the shape-transformable organohydrogel. (**e**–**g**) One-dimensional stretching and shape fixation of the shape-transformable organohydrogel. (**h**) Thermal imaging of the shape recovery process. (**i**–**l**) Two-dimensional stretching and shape recovery of the shape-transformable organohydrogel.

As shown in Figure 11, the shape-transformable organohydrogel exhibits foldability, where it can be folded into a more compact configuration, such as a cube. The organohydrogel retains this folded shape until it is heated (Figure 11c), at which point it transitions back to its original flat shape (Figure 11d, Supplementary Video S1). This demonstrates the organohydrogel’s shape-memory capability, as it can recover its original form after deformation.

In addition to folding, the shape-transformable organohydrogel can also hold a stretched state. The organohydrogel is stretched along one dimension after heating (Figure 11f). Although the external stretching force is removed, the organohydrogel remains in the stretched state after cooling (Figure 11g, top), demonstrating its ability to maintain deformation without external force. The organohydrogel returns to its original configuration upon heating (Figure 11g, bottom), confirming its shape-memory capability. Figure 11h shows the result of thermal imaging during deformation for recovery. The shape recovery is triggered at a temperature of around 40 °C, which is close to body temperature, and the transformation is completed within a few minutes (Supplementary Video S2). When the organohydrogel is heated, the gallium mesh transitions to a liquid state, allowing the stored stress in the organohydrogel to be released. This causes the organohydrogel to return to its original configuration.

In the case of 2D stretching, the organohydrogel was extended across two dimensions with the custom-made radial stretcher, as depicted in Figure 11i,j. The material maintained the stretched state until heating was applied (Figure 11k), after which the organohydrogel returned to its initial shape (Figure 11l, Supplementary Video S3), demonstrating its capability to handle multi-directional stretching and maintain shape fixity until activated by heat.

These observations highlight the organohydrogel’s ability to not only withstand various types of mechanical deformation but also recover its original shape, making it well-suited for applications that require reconfigurable and shape-memory materials.

## 3. Conclusions

In this work, we successfully developed a shape-transformable organohydrogel by incorporating a gallium mesh into a polyacrylamide-alginate matrix. The combination of gallium’s phase-change properties with the organohydrogel’s intrinsic flexibility allowed the material to undergo various deformations, including folding, 1D stretching, and 2D stretching while retaining its ability to recover its original shape upon heating. Our results demonstrate that the organohydrogel maintains its mechanical integrity, with the gallium providing structural support when solidified and allowing for flexibility when in its liquid state.

To address the challenge of maintaining the organohydrogel’s stretchability and flexibility during repeated heating and cooling cycles, we incorporated glycerol into the organohydrogel matrix. Glycerol’s anti-drying properties help retain moisture, ensuring the organohydrogel remains flexible and stretchable over time. Organohydrogels with glycerol concentrations of 30% or higher exhibited the best balance between moisture retention, flexibility, and mechanical strength, making them ideal for long-term applications where continuous deformation and shape retention are required.

These findings highlight the potential of this shape-transformable organohydrogel for use in applications that demand both reconfigurable structures and shape-memory capabilities. The material’s ability to deform and then recover its original shape upon heating makes it suitable for use in areas such as soft robotics, flexible electronics, and other dynamic systems that benefit from responsive materials. Furthermore, the universal nature of the gallium mesh approach enables this strategy to be adapted to other polymeric systems, expanding its potential beyond the hydrogel matrix used in this study.

Future research could explore the material’s use in more complex environments, including biocompatible and implantable devices, where shape retention, flexibility, and adaptability are critical. Additionally, investigating the optimization of polymer matrix and gallium mesh parameters, including the bonding between the hydrogel matrix and gallium mesh, could further improve the organohydrogel’s performance and broaden its applicability in advanced engineering and medical systems.

## 4. Materials and Methods

### 4.1. Materials

For PAAm-Alginate gel, Acrylamide (AAm, A8887, Sigma-Aldrich, St. Louis, MO, USA) was used as the monomer for the PAAm hydrogel network. N,N′-methylenebisacrylamide (MBAA, M2877, TCI, Tokyo, Japan) was used as the crosslinker. Sodium Alginate (A0205, TCI, Tokyo, Japan) was used as the monomer for the alginate hydrogel network. Ammonium persulfate (APS, A3678, Sigma-Aldrich, St. Louis, MO, USA) was used for curing organohydrogel substrates. APS functions as a thermal or photo-initiator. Glycerol (99.0%, Samchun, Seoul, Republic of Korea) was used for its anti-drying properties. Gallium (99.99%, Sigma-Aldrich, St. Louis, MO, USA) was used for the gallium mesh frame.

### 4.2. Synthesis of Stretchable Anti-Drying Organohydrogel

The stretchable hydrogel was synthesized by mixing linear copolymer alginate and covalently crosslinked PAAm [25]. Alginate solution (3 wt%) in a water (deionized)-glycerol mixture was prepared, where the glycerol content varied by weight (0, 10, 20, 30, 40, and 50 wt%). AAm was added to the alginate solution and mixed. The ratio of alginate to AAm was 1:8 by weight. When the AAm was fully dissolved, the mixture was mixed with 2 wt% MBAA (0.06% of the total weight of AAm). For the last, APS (0.75% of the total weight of AAm) was added to cure the hydrogel pre-gel solution. The mixing process was conducted using a planetary centrifugal mixer at 2000 r.p.m. (AR-100, Thinky Corporation, Tokyo, Japan). The cured hydrogel was prepared by UV radiation (XLite 300, OPAS, Taichung, Taiwan).

### 4.3. Fabrication of the Gallium Mesh Frame

To create the mesh structure, two layers of gallium frames (warp and weft layers) were prepared. A 100 μm thick polyethylene terephthalate (PET) film on a glass substrate was patterned using laser cutting. This PET-patterned film was used to create an Ecoflex (0050, Smooth-On, Macungie, PA, USA) mold with microchannels (channel width: 400 μm, height: 100 μm). The mold was covered with a PET release film, and liquid gallium was injected into the microchannels and solidified in a refrigerator, forming a rigid mesh with a thickness of 100 μm. The two layers of the gallium frame were aligned and partially heated with a heat gun to bond them to each other, forming the final mesh frame.

### 4.4. Mechanical Analysis of the Organohydrogels

Samples were prepared in a bar shape and tested on a universal testing machine (QC-548M2F, Cometech, Taichung, Taiwan) at a strain rate of 0.5 mm/min. Each sample was cured in a 2-mm-thick polyacrylate mold, which was prepared using laser cutting. For testing, the samples were clamped after a PET film was bonded to hydrogel at the clamping sites using a cyanoacrylate adhesive (Loctite 406, Henkel, Düsseldorf, Germany).

### 4.5. Shape Fixation and Recovery Test

Samples (15 mm × 15 mm × 4 mm) were used for the shape fixation and recovery test. The samples were initially heated in a 60 °C convection oven for 5 min to reach the softened state for manipulation. After heating, the sample was placed in an acrylic mold that has the desired angle. For the maximum angle, denoted as $\theta_{max}$, the sample was bent to a parallel configuration to achieve the highest possible bending angle (180°). The mold and sample were then transferred to a freezer for 5 min, to cool below transition temperature, thereby maintaining the deformed shape. Once the shape was fixed, the sample was removed from the mold and the fixed bending angle, $\theta_{fixed}$, was recorded. To evaluate the shape recovery, the sample was reheated to 60 °C for 5 min in the convection oven, and the angle ($\theta_{i}$) was measured after reheating.

### 4.6. Thermal Imaging of the Shape Recovery Process of a Shape-Transformable Organohydrogel

A sample stretched to 50% strain and fixed in its deformed state was prepared. The sample was placed on a 40 °C hotplate. To prevent the sample from adhering to the plate surface and obstructing its shrinking movement, a drop of 50% aqueous glycerol solution was placed underneath the sample. The shape recovery process was recorded using a thermal camera (A400, FLIR, Wilsonville, OR, USA), capturing the transition as the hydrogel returned to its original shape.

### 4.7. FTIR Analysis of the Organohydrogels

FTIR spectroscopy was performed using a FTIR spectrometer (VERTEX 80v, Bruker, Billerica, MA, USA) to investigate the chemical interactions and structural changes in the organohydrogel samples with varying glycerol concentrations (0%, 10%, 20%, 30%, 40%, and 50%). Spectra were recorded in the range of 4000–600 cm^−1^ at a resolution of 8 cm^−1^. Samples were prepared as thin films (thickness of approximately 200 μm) and analyzed using the transmission mode. Background spectra were collected prior to each measurement to account for atmospheric interference. Peak assignments were made to identify specific functional groups.

## Data Availability

The original contributions presented in the study are included in the article/Supplementary Material, further inquiries can be directed to the corresponding author.

## Supplementary Materials

The following supporting information can be downloaded at https://www.mdpi.com/article/10.3390/gels10120769/s1: Figure S1. FTIR spectra of organohydrogel samples with varying glycerol concentrations (0%, 10%, 20%, 30%, 40%, and 50%). The O-H stretching peak is observed at 3347 cm^−1^, which shifts to lower wavenumbers as the glycerol concentration increases, indicating enhanced hydrogen bonding within the polymer network. Peaks around 2937 cm^−1^ and 2884 cm^−1^ correspond to the C-H stretching vibrations, which become more pronounced with higher glycerol content. Peaks in the range of 1400–1600 cm^−1^ represent the carboxylate functional groups from alginate. The region below 1100 cm^−1^ exhibits peaks associated with C-O stretching and deformation vibrations. Figure S2. Schematic representation of the two-layer gallium mesh fabrication process. (Left) Weft layer (transverse channels) and warp layer (longitudinal channels) are separately fabricated using microchannel molds. The two layers are aligned perpendicular to each other. (Right) Final gallium mesh structure formed by bonding the warp and weft layers through partial heating. Figure S3. Images of fracture formation in the gallium-reinforced organohydrogel when stretched beyond 15% strain in the solid-state gallium phase. (Left) Photograph of the tensile testing setup showing fractures along the gallium mesh. (Right) Microscopic image of the gallium mesh within the organohydrogel highlighting the fractured regions (indicated by arrows). Figure S4. Cyclic stretching and recovery tests of the shape-transformable organohydrogel over 10 cycles. The organohydrogel was initially stretched to 100% strain in the liquid gallium state and subsequently cooled to solidify the gallium for shape fixation. Images display the organohydrogel in its stretched and recovered states for each cycle, demonstrating consistent shape recovery and minimal structural deformation throughout the 10 cycles. The results highlight the organohydrogel’s durability and reliable. Figure S5. Stress-strain curves of the shape-transformable organohydrogel after 10 cycles of thermal and mechanical cycling. The black curve represents the organohydrogel in the cooled state (solid gallium; E = 1400 kPa), while the red curve corresponds to the organohydrogel in the heated state (liquid gallium; E = 175 kPa). The results demonstrate that the organohydrogel retains its mechanical characteristics in both states even after repeated cycles, confirming its durability and consistent performance under cyclic deformation and thermal transitions. Video S1: Heat-induced unfolding of shape-transformable organohydrogel cube; Video S2: Thermal imaging of shape-transformable organohydrogel under heating; Video S3: Heat-induced shape recovery of a 2D-stretched organohydrogel.
