# Supplementary material for: Stretchable and Shape-Transformable Organohydrogel with Gallium Mesh Frame
Source: Gels. 2024 Nov 26;10(12):769. doi: 10.3390/gels10120769 (PMC11675981; doi:10.3390/gels10120769)
Supplement: Supplementary file 1 [file gels-10-00769-s001.zip › gels-3255923-supplementary-conversion-done.pdf]

gels

MDPI

Article

Stretchable and Shape-Transformable Organohydrogel with Gallium Mesh Frame

Mincheol Lee [1,*], Youngjin Choi [1], Young Min Bae [1], Seonghyeon Nam [2,3] and Kiyoung Shin [1]

[1] Electro-Medical Equipment Research Division, Korea Electrotechnology Research Institute (KERI), Ansan 15588, Republic of Korea
[2] School of Chemical and Biological Engineering, Institute of Chemical Processes, Seoul National University, Seoul 08826, Republic of Korea
[3] Center for Nanoparticle Research, Institute for Basic Science (IBS), Seoul 08826, Republic of Korea
* Correspondence: minckeri@keri.re.kr

Supplementary Materials

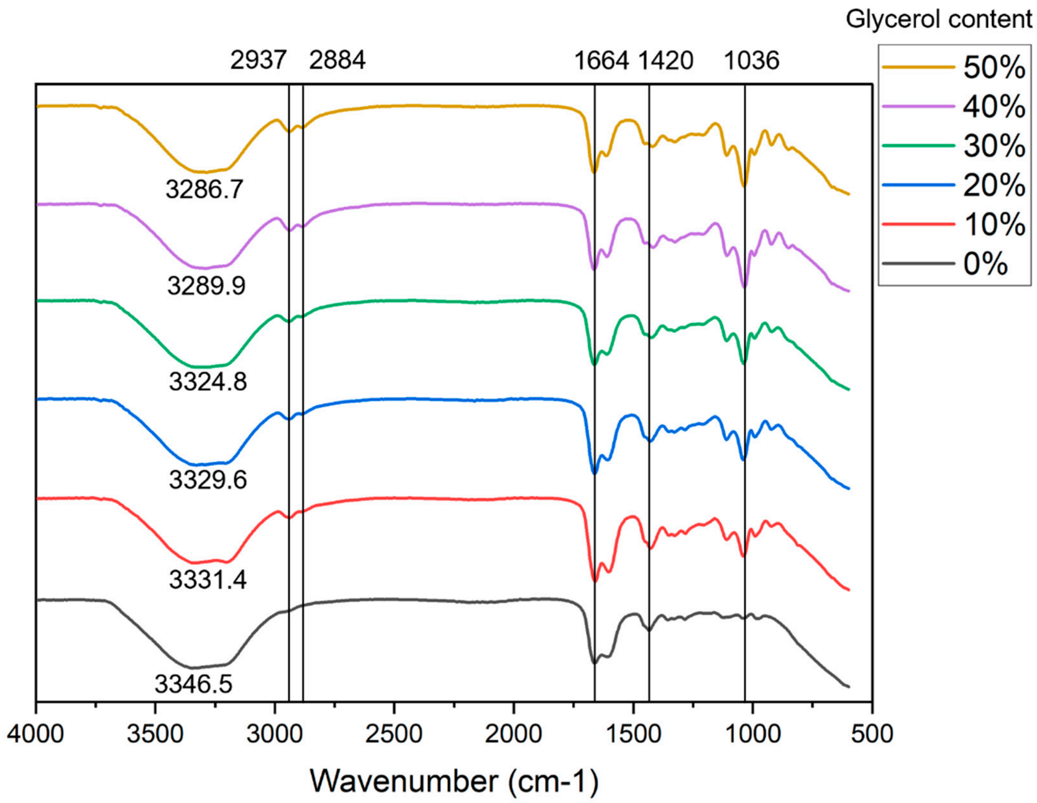

Supplementary Figure S1. FTIR spectra of organohydrogel samples with varying glycerol concentrations (0%, 10%, 20%, 30%, 40%, and 50%). The O-H stretching peak is observed at 3347 cm^{-1}, which shifts to lower wavenumbers as the glycerol concentration increases, indicating enhanced hydrogen bonding within the polymer network. Peaks around 2937 cm^{-1} and 2884 cm^{-1} correspond to the C-H stretching vibrations, which become more pronounced with higher glycerol content. Peaks in the range of 1400–1600 cm^{-1} represent the carboxylate functional groups from alginate. The region below 1100 cm^{-1} exhibits peaks associated with C-O stretching and deformation vibrations.

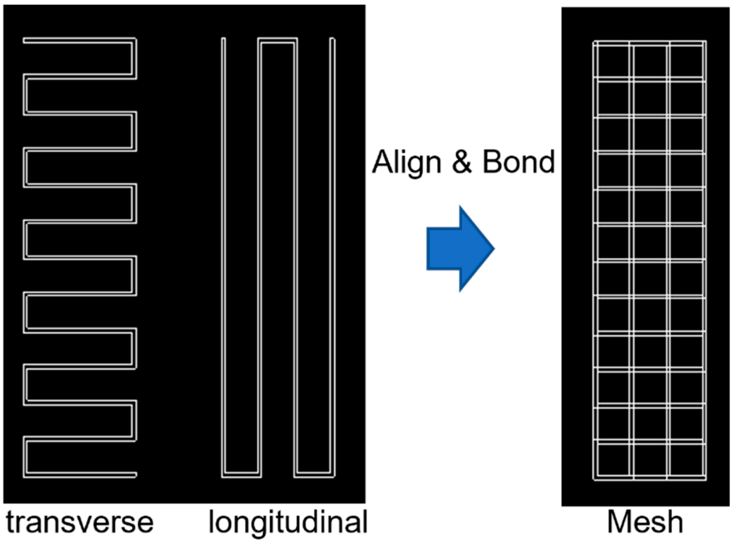

Supplementary Figure S2. Schematic representation of the two-layer gallium mesh fabrication process. (Left) Weft layer (transverse channels) and warp layer (longitudinal channels) are separately fabricated using microchannel molds. The two layers are aligned perpendicular to each other. (Right) Final gallium mesh structure formed by bonding the warp and weft layers through partial heating.

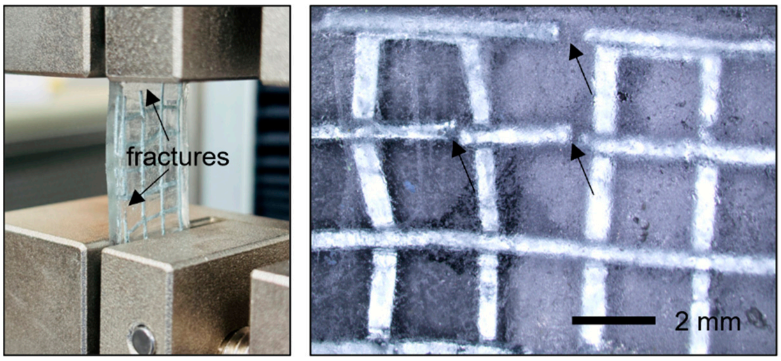

Supplementary Figure S3. Images of fracture formation in the gallium-reinforced organohydrogel when stretched beyond 15% strain in the solid-state gallium phase. (Left) Photograph of the tensile testing setup showing fractures along the gallium mesh. (Right) Microscopic image of the gallium mesh within the organohydrogel highlighting the fractured regions (indicated by arrows).

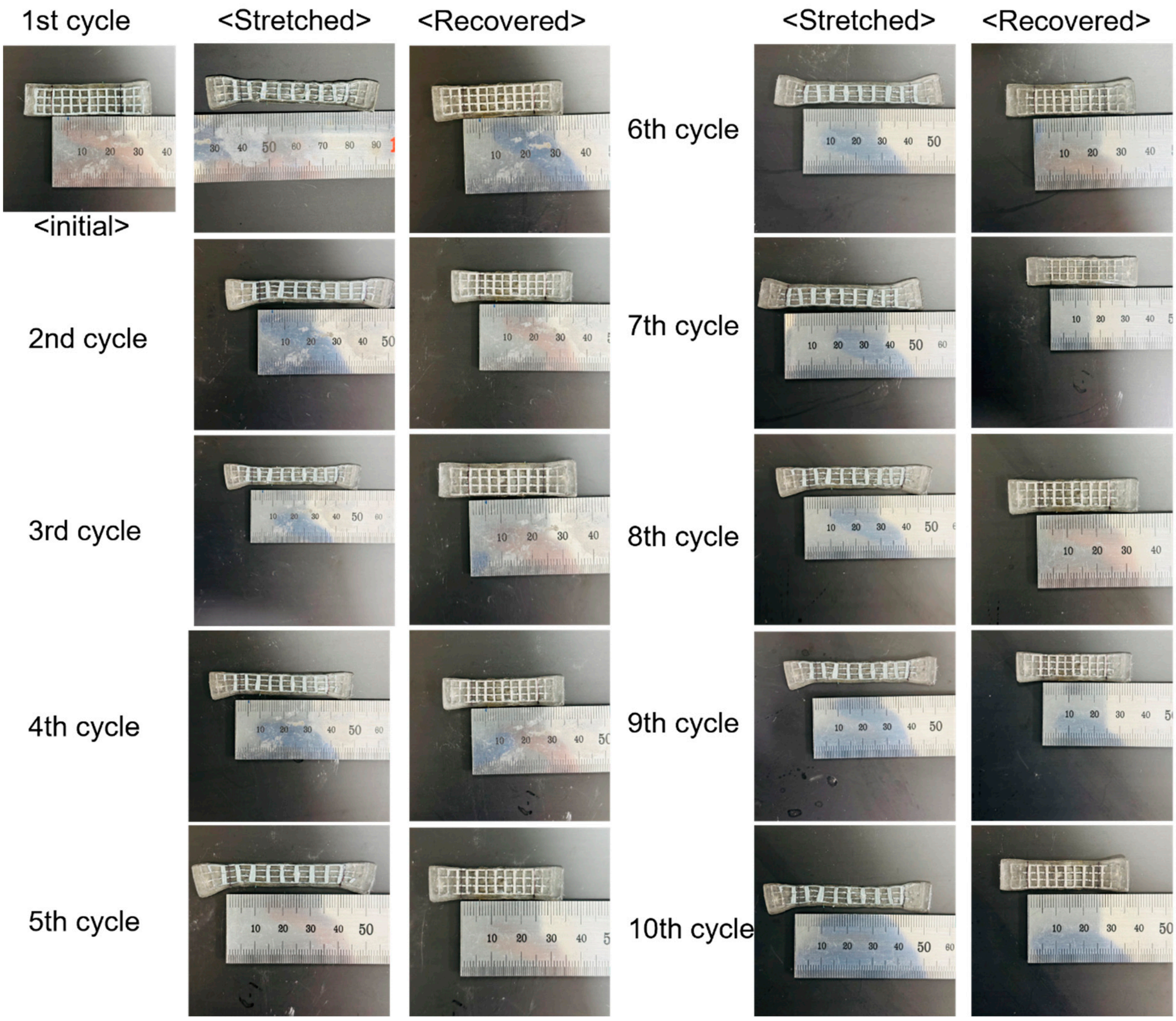

Supplementary Figure S4. Cyclic stretching and recovery tests of the shape-transformable organohydrogel over 10 cycles. The organohydrogel was initially stretched to 100% strain in the liquid gallium state and subsequently cooled to solidify the gallium for shape fixation. Images display the hydrogel in its stretched and recovered states for each cycle, demonstrating consistent shape recovery and minimal structural deformation throughout the 10 cycles. The results highlight the organohydrogel's durability and reliable

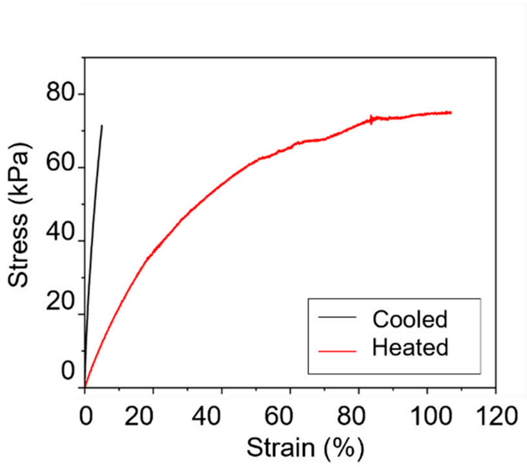

Supplementary Figure S5. Stress-strain curves of the shape-transformable organohydrogel after 10 cycles of thermal and mechanical cycling. The black curve represents the hydrogel in the cooled state (solid gallium; E= 1400kPa), while the red curve corresponds to the organohydrogel in the heated state (liquid gallium; E=175kPa). The results demonstrate that the organohydrogel retains its mechanical characteristics in both states even after repeated cycles, confirming its durability and consistent performance under cyclic deformation and thermal transitions.
